# Identification of Small Molecule Compounds for Pharmacological Chaperone Therapy of Aspartylglucosaminuria

**DOI:** 10.1038/srep37583

**Published:** 2016-11-23

**Authors:** Antje Banning, Christina Gülec, Juha Rouvinen, Steven J. Gray, Ritva Tikkanen

**Affiliations:** 1 Institute of Biochemistry, Medical Faculty, University of Giessen, Friedrichstrasse 24, 35392 Giessen, Germany; 2 Department of Chemistry, University of Eastern Finland, PO BOX 111, 80101 Joensuu, Finland; 3 Gene Therapy Center and Department of Ophthalmology, University of North Carolina, Chapel Hill, NC 27302, USA

## Abstract

Aspartylglucosaminuria (AGU) is a lysosomal storage disorder that is caused by genetic deficiency of the enzyme aspartylglucosaminidase (AGA) which is involved in glycoprotein degradation. AGU is a progressive disorder that results in severe mental retardation in early adulthood. No curative therapy is currently available for AGU. We have here characterized the consequences of a novel AGU mutation that results in Thr122Lys exchange in AGA, and compared this mutant form to one carrying the worldwide most common AGU mutation, AGU-Fin. We show that T122K mutated AGA is expressed in normal amounts and localized in lysosomes, but exhibits low AGA activity due to impaired processing of the precursor molecule into subunits. Coexpression of T122K with wildtype AGA results in processing of the precursor into subunits, implicating that the mutation causes a local misfolding that prevents the precursor from becoming processed. Similar data were obtained for the AGU-Fin mutant polypeptide. We have here also identified small chemical compounds that function as chemical or pharmacological chaperones for the mutant AGA. Treatment of patient fibroblasts with these compounds results in increased AGA activity and processing, implicating that these substances may be suitable for chaperone mediated therapy for AGU.

Aspartylglucosaminuria (AGU; OMIM 208400) is a recessive lysosomal storage disorder caused by mutations in the gene coding for aspartylglucosaminidase [AGA, *N*4-(β-*N*-Acetylglucosaminyl)-L-Asparaginase, EC 3.5.1.26]. AGU belongs to rare diseases, but it is enriched in Finland where most AGU patients have been described. Most AGU alleles in Finland carry the same mutation that results in an exchange of Cys163 to Ser (C163S), which is associated with a polymorphism Arg161Gln and designated as AGU-Fin. However, AGU cases detected elsewhere in the world exhibit their own unique mutations^1^,^2^,^3^. Impairment of AGA activity causes an accumulation of the AGA substrates, glycoasparagines, in lysosomes, and results in progressive mental decline of the patients. In the early adulthood, AGU patients are severely mentally retarded and show typical symptoms of AGU such as coarse facial features and skeletal abnormalities.

The AGA enzyme is synthesized as a single precursor molecule of 346 amino acids. After removal of the signal peptide, AGA precursor becomes N-glycosylated in two Asn residues^4^. Two precursor polypeptides dimerize soon after their synthesis, which results in an autocatalytic rearrangement and cleavage of the peptide bond between Asp205 and Thr206, giving rise to the active (αβ)_2_ tetrameric AGA. The catalytic function of AGA in glycoasparagine cleavage has been shown to involve the free α-amino group of the terminal residue Thr206 of the β subunit, placing AGA in the group of so-called N-terminal nucleophile hydrolases^5^,^6^. Thus, correct cleavage of the precursor into subunits is a prerequisite for the enzyme activity of AGA^7^. Most of the AGU mutations do not hit the active site of AGA, but rather affect the conformation of the precursor and thus prevent the cleavage into subunits. For example, AGU-Fin impairs the formation of an intramolecular disulfide bridge and thus destabilizes the enzyme structure, preventing the precursor cleavage into subunits^8^,^9^.

Currently, no treatment for AGU is available, although several preclinical studies aiming at enzyme replacement (ERT)^10^,^11^ or gene therapy^12^,^13^ have been published. Neither of these treatment options is expected to be available within the next few years, and there is thus a vast demand for an alternative therapy for AGU. The concept of pharmacological chaperone (PC) therapy has been emerging in the last years^14^,^15^,^16^. PC compounds are small molecules that bind into their protein targets and stabilize their native structures, helping mutated proteins to regain their biological function^17^,^18^. In some lysosomal storage disorders, first clinical trials have provided encouraging results. Since AGU mutations generally do not affect the active site of the enzyme directly, PC therapy might provide a non-invasive treatment option for AGU. We here identify potential small molecular substances that are capable of functioning as PCs for the mutated AGA enzyme, and show that these compounds result in increased AGA activity and improved lysosomal morphology in patient fibroblasts with two different mutations. These findings thus pave way for a PC-based therapy for AGU.

## Results

**Figure 1:**
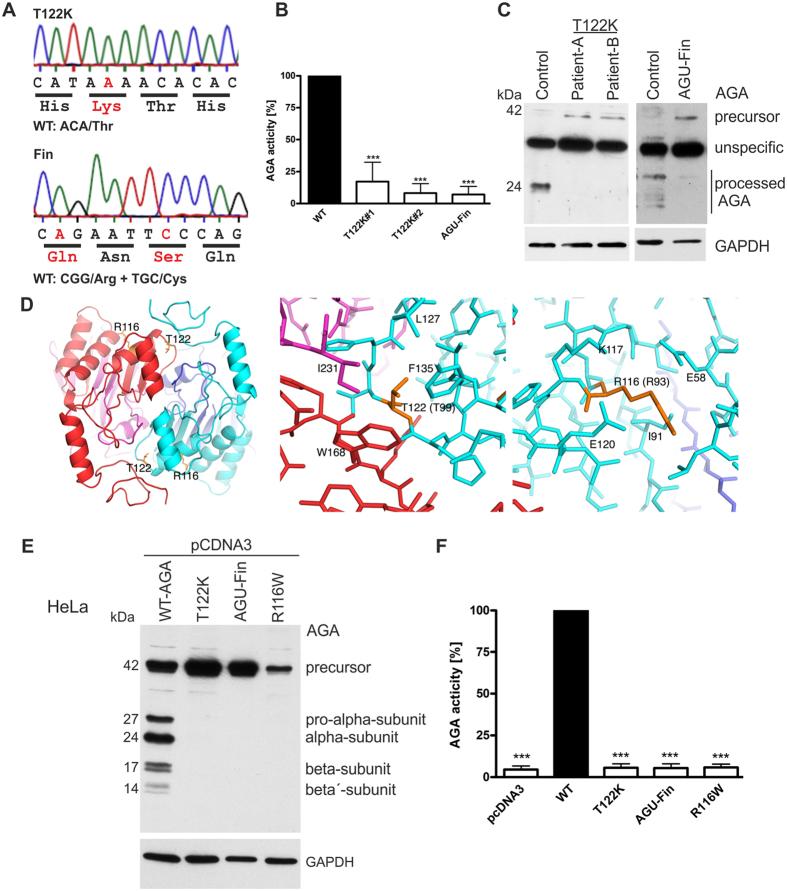
Characterization of the novel T122K aspartylglucosaminuria mutation. (**A**) Mutations that result in T122K and Arg161Gln plus Cys163Ser amino acid changes in AGU. Please note that Cys163Ser is the disease causing mutation, whereas Arg161Gln is a functionally neutral polymorphism. (**B**) AGA activity in control and AGU fibroblasts. N ≥ 7, shown as the mean of the data ± SD. Statistical analysis by One-Way Anova. (**C**) Processing of AGA in fibroblasts of AGU patients. (**D**) Localization of the mutated residues R116 and T122 in the structure of human AGA. The two αβ heterodimers are in cyan/blue and red/purple. (**E**) Processing of overexpressed, untagged AGA in HeLa cells. (**F**) AGA activity in cell lysates of transfected HeLa cells, N ≥ 10, shown as the mean of the data ± SD. Statistical analysis by One-Way Anova.

Two male US siblings were diagnosed with AGU, based on urinary excretion of glycoasparagines and typical symptoms of AGU. The clinical diagnosis was verified by genetic analysis, revealing a large genomic deletion in the *AGA* gene of maternal origin, whereas the paternal allele exhibited a single base exchange (c.365 C > A) in position 365 of the coding region of AGA. This results in exchange of Thr122 into Lys (T122K, Fig. 1A). The large deletion mutation in the maternal allele is predicted to result in the absence of expression of any AGA protein from this allele, reducing the total amount of expressed AGA protein by half in these patients. Measurement of the AGA enzyme activities in the patient fibroblasts with either the T122K or AGU-Fin mutation showed a significantly reduced enzyme activity, consistent with AGU (Fig. 1B).

To study the influence of the T122K mutation on AGA expression and processing, Western blot experiments with lysates of fibroblasts of the patients were performed. In T122K mutant cells, only the 42 kDa precursor AGA was detected, whereas control fibroblasts mainly exhibited the 24 kDa processed α subunit (Fig. 1C). A similar pattern as with the T122K mutant was observed in fibroblast lysates of an AGU patient who is homozygous for the AGU-Fin mutation (Fig. 1A+C). Please note that the polyclonal antibody used for the Western blots only poorly recognizes the β subunit.

To gain insight into the possible consequences of the T122K substitution, we studied the location of T122 in the three-dimensional structure of the (αβ)_2_ tetrameric human AGA^5^. T122 is located in the α polypeptide chain of AGA. It is buried on the interface between two αβ dimers, making hydrophobic contacts with the residues from the α and the β polypeptide chain of the other half of the tetramer (Fig. 1D). According to the crystal structure, the T122K exchange can be expected to result in changes in the conformations and interactions of the surrounding residues. Furthermore, a positively charged Lys in the hydrophobic core on the dimer-dimer interface is energetically less favorable. Thus, the T122K mutation is likely to have an impact on the assembly of tetrameric (αβ)_2_ AGA and, consequently, on the activation of the enzyme.

Recently, an AGU mutation causing an Arg116Trp (R116W) substitution has been described in three siblings of Turkish origin^1^. This residue is in the vicinity of T122 in the primary structure of α-chain, but the R116 side chain is located between two α-helices on the surface of AGA, making salt bridges with Glu58 and Glu120. The substitution of a positively charged residue with a bulky hydrophobic residue may cause changes in the conformation of the surrounding polypeptide chain, which may again affect correct oligomeric assembly and activation of the enzyme. Fibroblasts of AGU patients exhibiting the R116W mutation were, unfortunately, not available.

In order to verify the processing defect caused by AGU-Fin, T122K and R116W substitutions, the coding regions of these variants were cloned in an expression vector and the proteins were expressed in HeLa cells (Fig. 1E). The wildtype AGA enzyme showed the processed subunits (27/24 kDa α and 17/14 kDa β subunit), in addition to some unprocessed 42 kDa precursor due to overexpression. However, in cells expressing the three mutants, only the precursor polypeptide was detected. In addition, the R116W polypeptide was consistently expressed at a lower level than T122K and AGU-Fin precursors, implicating that this mutation may render the mutant AGA polypeptide unstable. Consistent with the Western Blot data, the AGA activities measured in the cells overexpressing the three mutants were at the level of that measured in mock transfected HeLa cells, whereas WT AGA expressing cells showed a significantly increased AGA activity (Fig. 1F). The residual enzyme activity measured for T122K and AGU-Fin in patient fibroblasts is not detectable in the overexpression system. This is probably due to the endogenous background activity in HeLa cells, but may also be partly due to overload of the cells due to the high expression levels, since the overexpressed WT AGA also exhibits a substantial amount of the unprocessed precursor.

**Figure 2:**
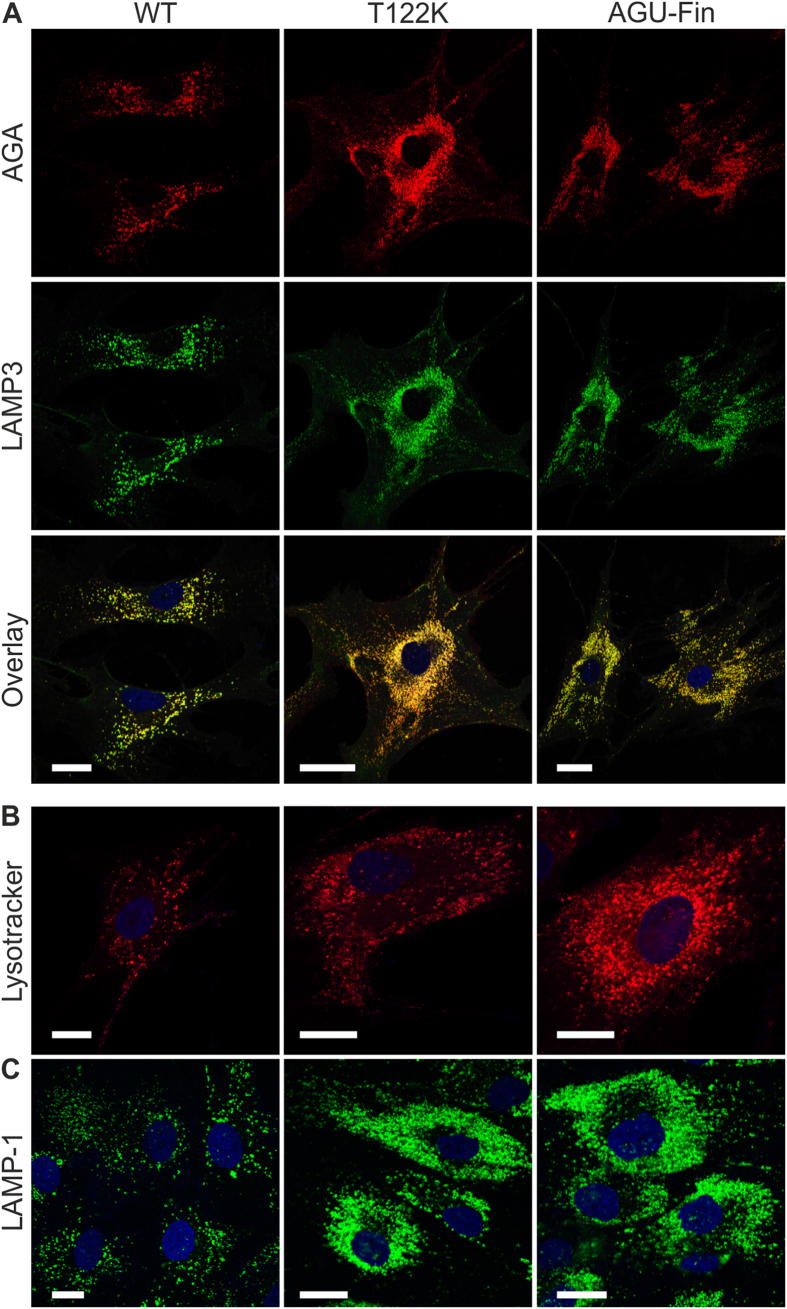
Lysosomal abnormalities in AGU fibroblasts. Staining of control, T122K and AGU-Fin fibroblasts for (**A**) AGA (red) and LAMP3 (green); (**B**) Lysotracker-red and (**C**): LAMP-1. Scale bar 20 μm.

**Figure 3:**
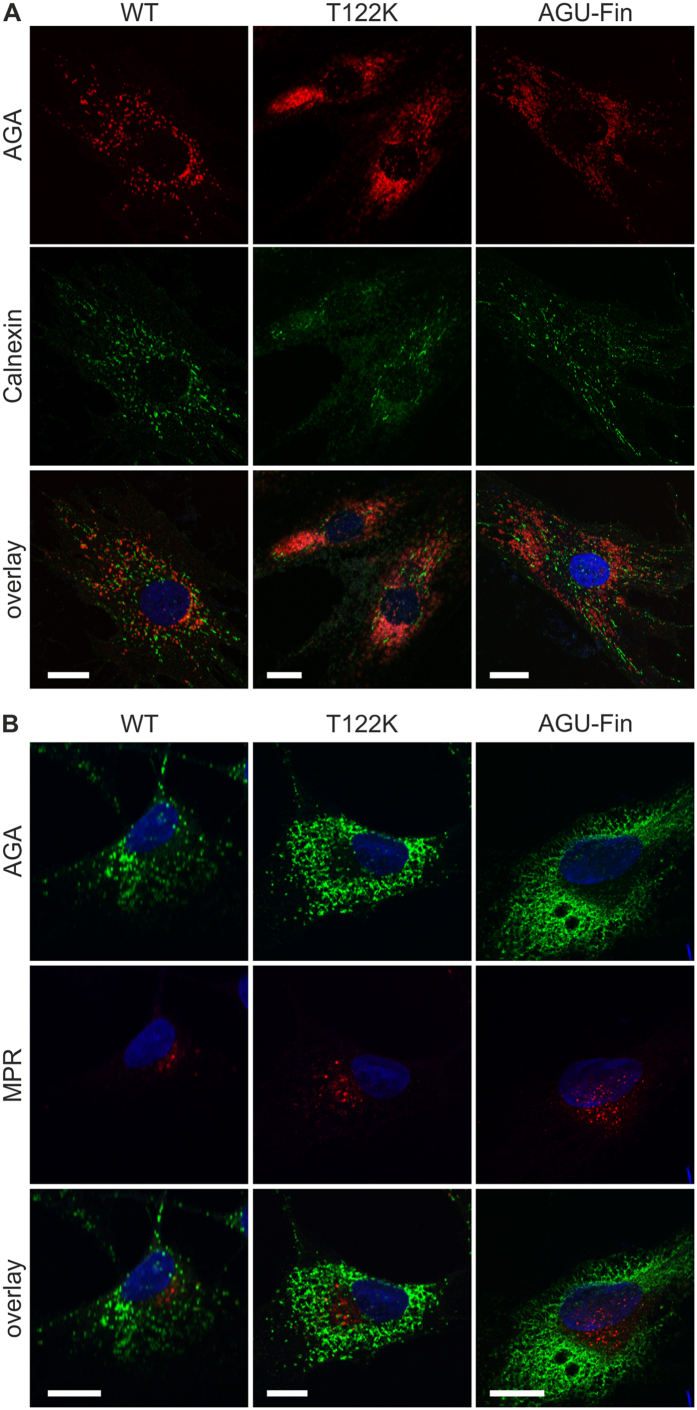
Mutated AGA proteins do not accumulate in the Golgi or ER. Staining of control, T122K and AGU-Fin fibroblasts for AGA (red) and (**A**) calnexin (green) or (**B**) cation-independent Mannose-6-phosphate receptor (MPR, green). Scale bar 20 μm.

To study the cellular localization of the mutant AGA polypeptides, control and patient fibroblasts were immunostained with antibodies against AGA and LAMP3/CD63, a lysosomal membrane protein. These stainings revealed a profound colocalization of AGA and LAMP3 in vesicular structures in both control and AGU fibroblasts (T122K and AGU-Fin, Fig. 2A). Consistent with a lysosomal storage disorder, the morphology of lysosomes in the AGU fibroblasts was altered as compared to control cells, as also revealed by Lysotracker (Fig. 2B) and LAMP-1 staining (Fig. 2C). Previous studies in overexpression systems have suggested that the AGU-Fin polypeptides accumulate in the ER and become degraded due to misfolding^19^,^20^. However, staining of AGU fibroblasts revealed only a very low degree of colocalization with calnexin, an ER marker (Fig. 3), suggesting that these mutations may impair the folded structure of AGA less than previously suggested, as they do not accumulate in the ER. Co-staining of the fibroblasts for AGA and cation-independent mannose-6-phosphate receptor (MPR300), which is localized mainly at the trans-Golgi network, also showed very little colocalization. Thus, our data show that in the patient fibroblasts, the T122K and AGU-Fin polypeptides localize mainly in lysosomes.

**Figure 4:**
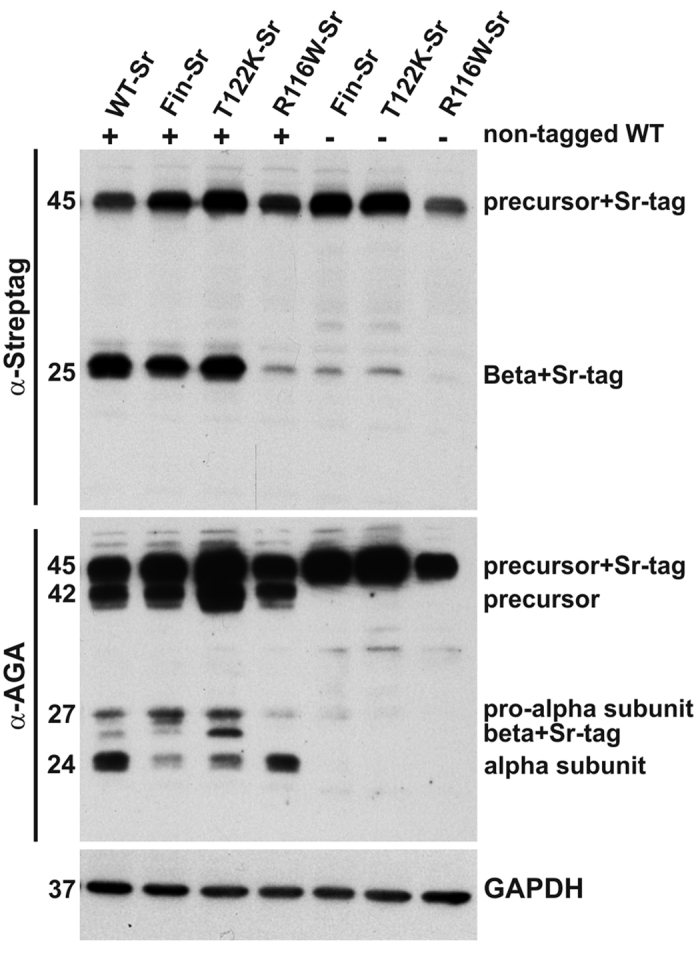
Coexpression of wildtype AGA results in enhanced activation of T122K and AGU-Fin mutated AGA proteins. Strep-tagged wildtype, AGU-Fin, T122K and R116W AGA proteins were coexpressed or not with the untagged wildtype AGA protein. Upper part: Western blot with the anti-Strep-tag antibody (recognizes tagged constructs), lower part: anti-AGA antibody (recognizes both tagged and untagged proteins). Please note that the Strep tag was localized in the C-terminus of the β subunit, and untagged α subunit is thus not detectable with the strep-tag antibody. Enhanced processing of AGU-Fin and T122K can be observed upon co-expression of the wildtype protein, whereas R116W remains as a precursor. N = 6.

The lysosomal localization of AGA is dependent on a conformational determinant that mediates its interaction with the phosphotransferase that generates the M6P signal, a prerequisite for the lysosomal targeting of AGA^21^. The activation of AGA occurs very rapidly after its folding, and requires that two precursor molecules form a dimer that then gives rise to the (αβ)_2_ heterotetrameric active structure. We speculated that the degree of misfolding caused by T122K and AGU-Fin mutations might be relatively moderate, as they are found in lysosomes (Fig. 2), although the mutant proteins remain in the precursor form. To test if dimerization of a WT precursor with the mutant precursor might be sufficient to induce the activation of the mutant half, we coexpressed an untagged WT AGA together with the C-terminally strep-tagged mutants T122K, AGU-Fin and R116W (Fig. 4). Specific detection of the mutant polypeptides was facilitated by an antibody against the strep-tag. When expressed alone, the mutant AGU proteins remained in the precursor form. In addition, T122K and AGU-Fin showed only a very minor band for the β subunit, which was not detectable in R116W transfected cells. R116W precursor was again detected in lower levels than the other two mutants. When untagged WT AGA was coexpressed with the mutant AGU polypeptides, T122K and AGU-Fin exhibited a substantial amount of processed polypeptide, implicating that their processing was indeed induced by codimerization with the WT precursor. Again, this processing was not detected with the R116W polypeptide, implicating a more serious folding defect in the case of this mutant.

**Figure 5:**
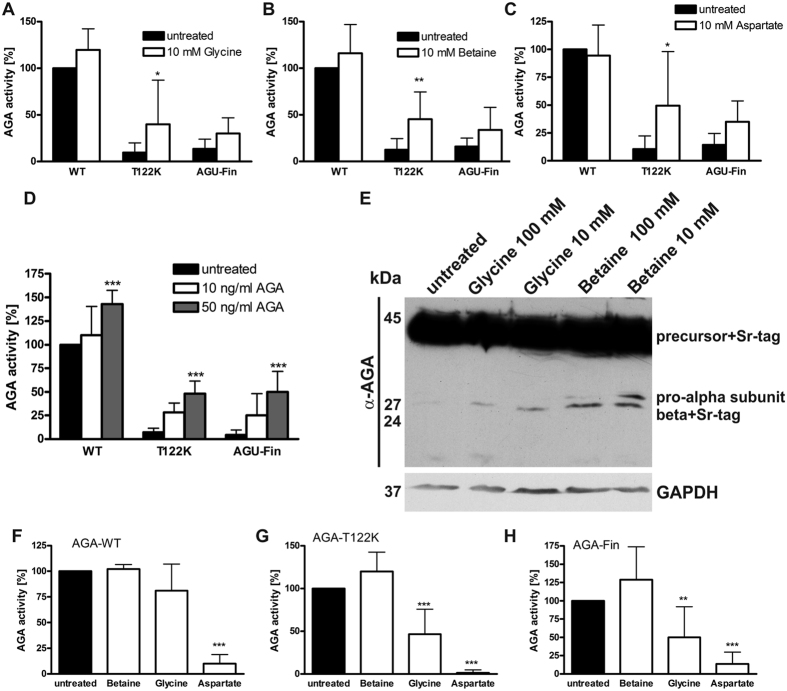
Chaperone treatment enhances AGA activity in AGU fibroblasts. (**A**–**C**) Wildtype, T122K and AGU-Fin fibroblasts were treated for 48 h with 10 mM chaperone substances, and the AGA activity was measured. (**A**) Glycine, (**B**) betaine, (**C**) Asp. (**D**) Activity increase by the chemical chaperones is comparable to that obtained by treatment (48 h) with 50 ng/ml recombinant human AGA. (**E**) HEK 293 T cells stably transfected with Strep-tagged T122K AGA were treated as indicated and the processing of AGA was detected by Western blot using anti-AGA antibodies. GAPDH was used as a loading control. (**F**–**H**) Treatment of purified, recombinant AGA enzymes with the chaperone substances. (**F**): Wildtype AGA, (**G**) T122K AGA, (**H**): AGU-Fin AGA. (**A**–**C**) & (**F**–**H**) N ≥ 6, graphs show the mean of the data ± SD. Statistical analysis by Two-Way Anova.

Pharmacological chaperones (PC) are small chemical compounds that bind to their respective protein targets and can facilitate their correct folding^16^,^17^,^18^,^22^. In the case of enzymes, PC substances frequently show a similar chemical structure as the substrates or inhibitors of these enzymes. We rationalized that such PC-like compounds might also facilitate the activation of the mutant AGA polypeptides by improving the correct folding of the mutant proteins and thus promoting the autocatalytic activation. Potential PC compounds were identified either on the basis of their structural features (betaine, Asp) or based on their putative function as enzyme inhibitors (Gly). When control or AGU patient fibroblasts were treated with 10 mM compounds (Fig. 5A–C), a substantial increase of AGA enzyme activity was observed with all three compounds, with significant data for T122K mutation. However, due to the large variation in the degree of AGA activation, the data did not reach significance in the case of AGU-Fin. The observed increase in AGA enzyme activity upon PC treatment was comparable to that obtained upon incubation with 50 ng/ml recombinant WT AGA enzyme (Fig. 5D). To show that the PC induced activity increase was due to enhanced AGA activation, HEK 293 T cells were stably transfected with Strep-tagged T122K AGA, treated or not with Gly or betaine, and the cells lysates were subjected to Western blot with an anti-AGA antibody. Treatment with Gly or betaine resulted in appearance of the processed subunits, demonstrating increased processing into subunits due to PC treatment (Fig. 5E). Interestingly, 10 mM concentration appeared to induce a greater degree of AGA activation than 100 mM. This may suggest that there is a certain therapeutic window where the substances are the most effective, and exceeding the effective range results in lower AGA activation.

Strep-tagged, recombinantly expressed, purified AGA proteins were incubated with the potential PC compounds to test if they would be capable of inducing the activation of the purified polypeptides (Fig. 5F–H). Betaine did not show any effect on the WT AGA polypeptide, whereas Gly and Asp substantially inhibited its activity, consistent with their role as AGA inhibitors. Both T122K and AGU-Fin polypeptides show some residual enzyme activity which was to a minor degree enhanced by betaine, whereas Gly and especially Asp resulted in a significant suppression of this residual activity (Fig. 5F–H).

**Figure 6:**
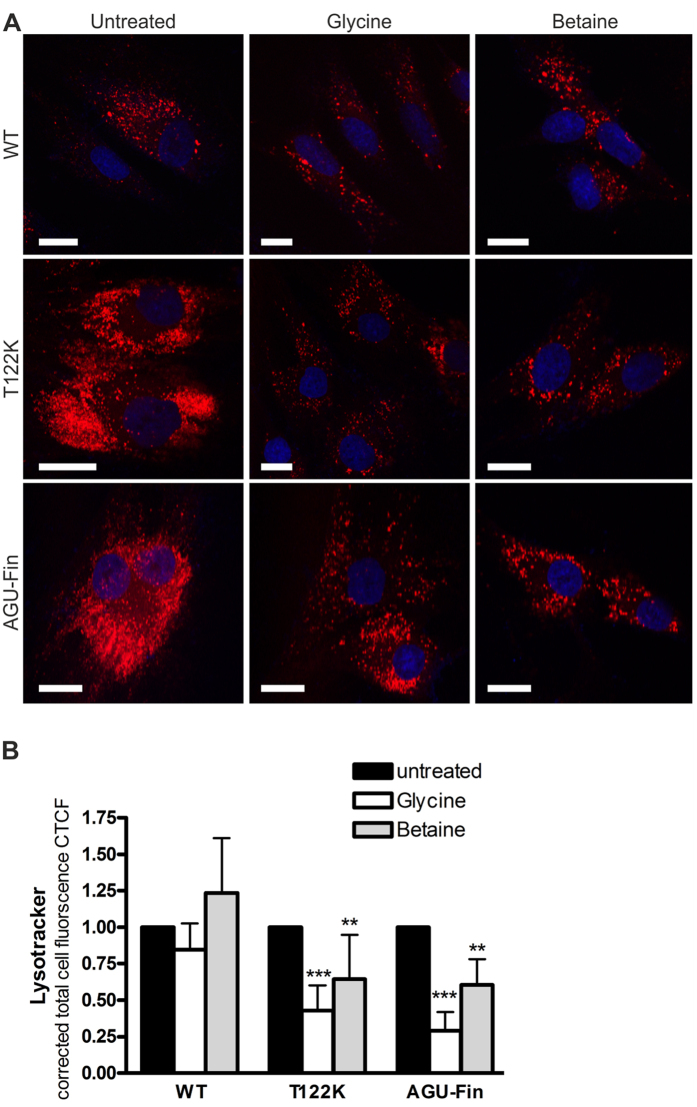
Treatment with chaperone substances ameliorates lysosomal pathology in AGU fibroblasts. (**A**) Lysotracker-red staining of wildtype, T122K and AGU-Fin fibroblasts treated with Gly or betaine. (**B**) Quantification of the Lysotracker intensity after chaperone treatment. At least 60 cells from 4 experiments were quantified. The data are shown as mean ± SD, statistical analysis by Two-Way Anova.

Impairment of lysosomal morphology is a hallmark of lysosomal storage diseases. We therefore studied the effect of Gly and betaine treatment on lysosomes using Lysotracker staining in control and AGU fibroblasts (Fig. 6A). Lysotracker staining intensity was significantly reduced in T122K and AGU-Fin fibroblasts by both Gly and betaine (Fig. 6B), implicating that the increase in AGA activity due to the PC compounds is sufficient to improve the lysosomal morphology in AGU fibroblasts.

## Discussion

In this study, we show that specific compounds that are likely to act as pharmacological chaperones can facilitate the activation and cleavage into subunits of the mutated AGA enzyme, and thus provide a potential therapy for AGU. Currently, curative therapy for AGU is not available due to diverse reasons. Already in the 1990’s, bone marrow transplantation (BMT) was carried out with some patients, but the therapeutic effect remained very benign as compared to the side effects of BMT^23^,^24^. In addition, the two existing AGU mouse models^25^,^26^ have been subjected to therapy trials using ERT or gene therapy^10^,^11^,^12^,^13^, but these studies have so far not led to clinical trials with patients, despite their beneficial effects. ERT as a potential AGU therapy has been hampered by difficulties to produce sufficient amounts of recombinant AGA enzyme for therapy of human patients. Development of gene therapy approaches has experienced a revival in the last few years due to development of safer and more effective viral vectors^27^, but it will still take a substantial amount of time until this therapy option is available for AGU.

Our data show that both T122K and AGU-Fin AGA enzyme variants most likely exhibit a relatively local folding defect that neither completely impairs the structure, nor results in severe destabilization and degradation of the mutant enzyme. These findings are somewhat contradictory to what has been proposed for the AGU-Fin mutated AGA, which has been suggested to become rapidly degraded after synthesis in the ER^19^,^28^. However, these previous studies have been based on overexpression of the mutated enzyme mainly in chinese hamster ovarian cells, which may result in ER overload and unfolded protein response, facilitating the degradation of the AGU-Fin mutant precursors. We here show that endogenous T122K and AGU-Fin AGA in patient fibroblasts do not colocalize with the ER marker calnexin, whereas a high degree of colocalization was observed with lysosomal markers. However, it is likely that the misfolding induced by T122K mutation is milder than that of AGU-Fin which impairs the formation of an intramolecular disulfide bond^20^. This would also be consistent with the data that the PC-like compounds used here showed a higher increase of enzyme activity in the case of T122K than AGU-Fin.

The autocatalytic processing of the AGA precursor is based on an intramolecular rearrangement of the scissile bond between Asp205 and Thr206. In the AGA precursor molecule, this bond has been suggested to be under a conformational strain that is released upon cleavage to the subunits^7^. In the case of the mutant AGA precursors, this rearrangement is severely reduced, preventing the cleavage into subunits. It has been shown that dimerization of two precursor molecules is a prerequisite for the cleavage, and some AGU mutations in the dimer interface prevent dimerization and thus activation^2^,^29^,^30^. However, this appears not to be the case with T122K and AGU-Fin, which both become processed into subunits when coexpressed with the wildtype AGA. This suggests that the wildtype AGA half in the precursor dimer is able to act as a folding scaffold that stabilizes the mutant precursor partner, facilitating its correct processing into subunits.

The present study and previous findings have identified AGU as a conformational disease that is largely based on local misfolding of the AGA enzyme, whereas only few mutations directly hit the active site of the enzyme. Thus, the main cause of the missing AGA activity on the case of T122K, AGU-Fin and possibly other AGU mutants is the deficient precursor processing. Therefore, conformational rescue by means of chemical substances that bind to the mutant AGA precursor, stabilizing its structure and allowing correct folding and processing, appeared feasible. Indeed, the PC-like substances identified here are capable of enhancing both the processing and activity of AGA, and ameliorate the lysosomal pathology in AGU patient fibroblasts. It has been shown previously that Gly and Asp are inhibitors of AGA^31^,^32^ and thus capable of directly binding to the active site of AGA. In the case of AGA precursor, a surface loop partially blocks the catalytic center, thus preventing substrate binding. However, the crystal structure of *Flavobacterium* precursor AGA revealed that glycine was still able to bind to the catalytic site^33^. Furthermore, Gly has been shown to enhance the processing of human asparaginase 3, the structure of which is highly similar to human AGA and which undergoes a similar autoprocessing^34^. Therefore, Gly and Asp can be considered as true pharmacological chaperones that directly interact with their target molecule and promote the folding of the mutant enzymes, thus enhancing their activation. Both substances were capable of increasing the AGA activity in AGU fibroblasts, but the use of especially Asp in therapy might be limited due to the fact that it significantly inhibited the AGA enzyme activity when purified recombinant WT, T122K and AGU-Fin AGA were treated with Asp. A more moderate but significant inhibition was observed with Gly, raising the question about the feasibility of Gly for AGU therapy.

The molecular mechanism of the enhancing effect of betaine (trimethyl-Gly) on AGA enzyme activity is not completely clear. Betaine is a small ligand and shows structural similarity with Gly. According to the crystal structures, there could be enough space to accommodate betaine in the active site, but we cannot rule out an indirect mechanism for the AGA enzyme activation. Betaine is a natural substance involved in human physiology and metabolism in various ways (reviewed in^35^). It has been shown to function as an osmolyte and lipotrope, preventing accumulation of lipids in the liver and probably protecting against oxidative stress. In addition, betaine is an important methyl group donor whose metabolism intersects with choline and various amino acids. Thus, betaine might enhance AGA activity in AGU fibroblasts by generally improving the metabolic/osmotic state of the cells, relieving ER or lysosomal stress and thus allowing improved AGA folding. This is consistent with our observations that although betaine resulted in a significant increase in AGA activity in fibroblasts, it only enhanced AGA activity of the purified recombinant T122K protein to a very moderate degree, suggesting an indirect mode of action. Furthermore, the observed degree of precursor processing into subunits (Fig. 5E) does not fully correlate with the increase in the enzyme activity. This could be due to the fact that the PC compounds apparently also improve the lysosomal morphology (Fig. 6), and may increase the enzyme activity indirectly, in addition to their effect on precursor activation, due to improved conditions in lysosomes.

Betaine (trade name Cystadane) is currently in clinical use for the therapy of homocysteinuria, and it is under clinical trials for peroxisomal disorders of the Zellweger spectrum. This disease is caused by mutations in the genes encoding for peroxisomal pexin (PEX) proteins. The PEX1 protein is normally involved in peroxisomal import. It was shown that betaine improved peroxisomal import of various proteins in fibroblasts exhibiting mutant PEX1 protein with G843D substitution^36^. The G843D PEX1 substitution is associated with a mild form of the disease, and its function can be partially rescued by expression of its binding partner PEX6, indicating that a conformational rescue takes place, similarly to what we here observed upon coexpression of T122K and AGU-Fin with the wildtype AGA. In addition, it has been shown that in primary hyperoxaluria, betaine is able to enhance the activity of alanine:glyoxylate transferase in cell culture^37^, and clinical studies are also on the way for this disease.

The present study paves way to a chaperone mediated therapy for AGU. Although the enzyme activity in AGU fibroblasts after betaine treatment does not reach the level of healthy fibroblasts, it is close to the activity that could be expected in cells of a carrier (50%), who would not exhibit any symptoms of the disease. Furthermore, it is known that even a lower degree of residual lysosomal enzyme activity can result in amelioration of disease symptoms in lysosomal storage disorders^38^. In accordance, we observed a clear improvement of lysosomal pathology upon treatment of AGU fibroblasts with betaine. We could also show that treatment of cells overexpressing the T122K mutant AGA with betaine or Gly resulted in increased processing into subunits, supporting our model for increased mutant AGA activation due to PC treatment.

The existing AGU mouse models are not suitable for testing of the PC compounds, since they are based on ablation of the AGA gene by targeted exon disruption, so that no protein is produced^25^,^26^. Therefore, the next step would be to test the substances, especially betaine, in AGU patients. Since betaine has already been used for pediatric patients, plenty of safety and dosage data are available, which will facilitate the therapeutic trial for AGU. However, it is not quite clear how efficiently betaine penetrates the blood-brain barrier, and it is unclear if it exerts an effect on the central nervous system. Therefore, further PC substances for AGU mutants should be identified in the future by screening of chemical libraries. In addition, additional AGU point mutations should be tested for the therapeutic benefit with our PC compounds.

## Materials and Methods

### Cell culture

HeLa (human cervix carcinoma cells) and HEK293T (human embryonic kidney cells) cells were cultured in Dulbecco’s modified Eagle’s medium (DMEM) with high glucose, supplemented with 10% fetal calf serum (FCS), 1% penicillin/streptomycin (both from Gibco, Thermo Fisher Scientific, Germany). Primary skin fibroblasts with AGU-Fin mutation (Catalog ID: GM00568) were obtained from Coriell Institute for Medical Research (Camden, NJ, USA). Primary skin fibroblasts with T122K mutation were obtained from a skin biopsy of two US American siblings. Informed consent of the donors’ parents was obtained. Immortalized, hTERT-transformed normal human skin fibroblasts were as described^39^. All fibroblasts were cultured in DMEM with high glucose, supplemented with 10% FCS, 1% penicillin/streptomycin, 1% non-essential amino acids and 1% sodium pyruvate. All cells were grown at 8% CO_2_ and 37 °C.

### Antibodies

A rabbit polyclonal antibody (Enogene, Aachen, Germany) was used to detect AGA in Western blots. A mouse monoclonal antibody against GAPDH was purchased from Abcam (Cambridge, UK). Strep-tagged AGA protein was detected with a mouse monoclonal antibody (Novagen, Darmstadt, Germany). Primary antibodies for immunofluorescence stainings were: a rabbit antiserum against human AGA^4^, a mouse monoclonal calnexin antibody (sc-23954, Santa Cruz Biotechnology), a mouse monoclonal LAMP-3 antibody (sc-5275, Santa Cruz), a polyclonal rabbit LAMP-1 antibody (Abcam), a monoclonal mouse mannose-6-phosphate receptor (MPR300) antibody^40^.

### Plasmids and Transfection

AGA cDNAs from HeLa cells (wildtype AGA), from AGU-Fin and T122K-fibroblasts were amplified by PCR and cloned into pcDNA3 (Invitrogen, Thermo Fisher Scientific, Karlsruhe, Germany) using BamHI and XhoI and by XbaI and BamHI into pExpr-IBA103 (IBA, Göttingen, Germany) with a C-terminal Strep-tag. Primer sequences were:

AGA-pcDNA3-fwd: 5′-CTATAGGATCC ATGGCGCGGAAGTCGAACTTG-3′

AGA-pcDNA3-rev: 5′-CTATA CTCGAG TTA GATGCAGTCCACTTTTTCC-3′

AGA-IBA-fwd: 5′-CTATATCTAGA ATGGCGCGGAAGTCGAACTTG-3′

AGA-IBA-rev: 5′-CTATAGGATCC GATGCAGTCCACTTTTTCCTC-3′.

R116W amino acid substitution was produced by site-directed mutagenesis of the WT AGA construct. Correctness of all constructs was verified by sequencing.

24 h prior to transfection, the cells were seeded onto 12-well plates. For transfections, 400 ng of expression plasmids (either 400 ng in case of a single plasmid or 2 × 200 ng for cotransfection of two plasmids) were transfected using MACSfectin™ (Miltenyi Biotec, Bergisch Gladbach, Germany) according to the manufacturer’s protocol. After 24 h, cells were transferred onto 6-well plates and harvested 48 h post transfection in lysis buffer (50 mM Tris pH 7.4, 150 mM NaCl, 2 mM EDTA, 1% NP-40), supplemented with protease inhibitor cocktail for 30 min on ice. Protein concentrations were determined according to Bradford. Lysates were used for Western Blot.

### Purification of recombinant AGA proteins

HEK293T cells were stably transfected with AGA-pExprIBA103 constructs (wildtype, AGU-Fin, T122K). For selection with zeocin, empty pTER+ plasmid was cotransfected. For protein purification, confluent cells on 15 cm dishes were cultured in medium without FCS for 48 h. 100 ml of medium was concentrated to less than 2 ml using Amicon concentrators. AGA proteins were purified using gravity flow strep-Tactin®-superflow columns according to the manufacturer’s protocol (IBA GmbH, Göttingen, Germany).

### Treatment with recombinant protein and PCT compounds

Fibroblasts or transfected HEK 293 T cells were seeded into 6-well plates and treated with recombinant AGA (10–50 ng/ml) or 10 mM betaine (Sigma-Aldrich, Taufkirchen, Germany), glycine (Roth, Karlsruhe, Germany) or aspartic acid (Roth) for 48 h. All PCT substances were prepared as 1 M stock solutions in water. Cells were lysed as described above, and the lysates were used for enzyme activity measurements. For treating recombinant proteins, 5 μl of protein eluate was mixed with 40 μl folding buffer (100 mM NaCl, 0.1% Triton X-100, pH 4.5) and 5 μl 1 M substance and incubated for 3 h at 4 °C.

### Enzyme activity measurements

AGA activity was measured fluorimetrically according to Voznyi *et al*.^41^ with some modifications. Reaction mixtures consisted of 10 μl cell lysate (blank: lysis buffer) and 20 μl 50 μM Asp-AMC (L-Aspartic acid β-(7-amido-4-methylcoumarin) in McIlvain’s phosphate-citrate buffer pH 6.5. Samples were incubated for 24 h at 37 °C, and the reaction was terminated by the addition of 200 μl McIlvain’s buffer pH 4.5. A standard curve containing 10 μl of 7-Amino-4-methylcoumarin (AMC, Sigma-Aldrich, Taufkirchen, Germany) ranging from 0–20 μM AMC and 20 μl of 50 μM Asp-AMC was measured in parallel. All samples were measured in triplicates with a Tecan Infinite M200 plate reader using 355 nm excitation and 450 nm emission wavelengths, respectively. AGA activity was expressed as μmol AMC/mg protein.

### Immunofluorescence

Cells were seeded on glass coverslips and cultured for at least 2 days in the presence or absence of 10 mM PC compound. For staining of acidic compartments, cells were incubated with 5 μM Lysotracker-Red (Invitrogen) in cell culture medium for 30 min. All cells were fixed in 4% paraformaldehyde for 10 min at room temperature. Before antibody staining, cells were permeabilized with 0.1% digitonin and 1% BSA in PBS. Stainings with primary and secondary antibodies were carried out for 1 h at room temperature in PBS/1% BSA. Samples were analyzed with a Zeiss LSM710 Confocal Laser Scanning Microscope (Carl Zeiss, Oberkochen, Germany). Fluorescence of at least 15 cells per experiment was measured using ImageJ.

### Statistical analysis

All experiments were performed at least four times. Data are expressed as mean ± SD. Statistical comparisons between groups were made using Student’s *t*-tests, 1way or 2way ANOVA, as appropriate (GraphPad Prism 5, San Diego, CA, USA). Values of *p* < 0.05 were considered significant (*) while values of *p* < 0.01 were considered very significant (**) and *p* < 0.001 extremely significant (***).

### Electronic manipulation of images

The fluorescence images have in some cases as a whole been subjected to contrast or brightness adjustments. No other manipulations have been performed unless otherwise stated.

### Ethical statement

Human fibroblasts were obtained after a written informed consent of patients’ parents at Greenwood Genetics, South Carolina, USA. The experimental protocol was approved by Self-Regional Healthcare in Greenwood, SC, USA (IRB permission number 75). All experimental procedures were performed according to the guidelines of the Declaration of Helsinki.

## Additional Information

**How to cite this article**: Banning, A. *et al*. Identification of Small Molecule Compounds for Pharmacological Chaperone Therapy of Aspartylglucosaminuria. *Sci. Rep.* **6**, 37583; doi: 10.1038/srep37583 (2016).

**Publisher's note:** Springer Nature remains neutral with regard to jurisdictional claims in published maps and institutional affiliations.
